# Emerging hallmarks and the rise of complexities and heterogeneity of tumor

**DOI:** 10.1016/j.bbrep.2025.102347

**Published:** 2025-11-09

**Authors:** Hasmiq L. Arora, Gopinath Sekar, Anushka Phadnis, Anjali Bahot, Dhanashree Bomle, Vaidehi Patel, Jayanta K. Pal, Sachin C. Sarode, Nilesh Kumar Sharma

**Affiliations:** a Cancer and Translational Research Lab, Dr. D.Y. Patil Biotechnology and Bioinformatics Institute, Dr. D.Y. Patil Vidyapeeth, Pune, Maharashtra, 411033, India; b Dr. D.Y. Patil Vidyapeeth, Pune, Maharashtra, India

**Keywords:** Tumor hallmarks, Epigenetic alteration, Metabolic reprogramming, Immune evasion

## Abstract

Cancer is known for its complexities and heterogeneity due to genetic, epigenetic, known, and unknown environmental components. From time to time, incremental viewpoints on the expanding landscape of tumor hallmarks, including sustained proliferation, evasion of cell death, immune evasion, metabolic reprogramming, and the metabolic-epigenomic-immune axis, are presented. Unifying old and new tumor hallmarks may offer progressive platforms for new diagnostic, therapeutic, and monitoring approaches to therapy responses. Here, we strive to present an updated framework at intracellular, cellular, intercellular, and extracellular levels, assisted by emerging technologies such as OMICS and super-resolution imaging technologies. This review emphasizes that understanding emerging tumor hallmarks may help reveal the dynamics and heterogeneity of tumors. This could lead to sustainable diagnosis, prognosis, and therapeutic management, including precision and personalized approaches for cancer patients. This review presents a unified hierarchical model that connects classical and emerging tumor hallmarks through AI-powered multi-omics integration, emphasizing its conceptual innovation and translational potential in advancing precision oncology.

## Highlights

• Expands tumor hallmark view across cellular, intracellular, intercellular and extracellular frameworks.
• Critiques current hallmark framework and its limitations.
• Proposes refined segregation to capture cancer complexity.
• Explores tumor evolution, therapy resistance, and adaptation.
• Discuss prevention, diagnosis, prognosis, and therapies in the contexts of expanding tumor hallmarks.

## 1 Introduction

Cancer is seen as one of the leading contributors to global mortality, with 35 million new cases projected by 2050 [[1], [2], [3]]. Deaths due to cancer could be attributed to many factors, including genetic, epigenetic, environmental, and also the evolving attributes of cancer cells. The acquisition of functional capacities during the transition from normal to neoplastic development stages is ascribed to many critical traits known as tumor hallmarks [4,5]. Tumorigenesis is a highly intricate and multi-faceted process that enables cancer cells to achieve various hallmarks including sustained growth, uncontrolled proliferation, and invasiveness [[5], [6], [7]]. Hanahan and Weinberg's hallmark framework defined core traits like sustained proliferation and evasion of apoptosis. Recent advances include metabolic dysregulation, immune evasion, and epigenetic plasticity, reflecting the disease's adaptive complexity.

While this traditional framework is the foundation of cancer biology, there is a scope to explore ways that can describe the evolving functional and structural heterogeneity of tumors. Cancer evolves by a series of adaptive changes, with hallmark traits being acquired incrementally and dynamically over time at every stage. The existing conventional hallmarks denote sustained proliferation, evasion of growth signals, resistance to cell death, replicative immortality, angiogenesis, invasiveness, and metastasis [5].

Recent advances have supported the emerging tumor hallmarks such as the reprogramming of metabolism, epigenetic remodeling, and emergence of cancer-supporting immune cells [[6], [7], [8], [9]]. By systematically understanding each hallmark, clinicians and researchers can discover new therapeutic targets, anticipate resistance mechanisms, and develop personalized medicine approaches that enable tailored treatment for different types of cancer [[8], [9], [10], [11]].

Also, understanding tumor hallmarks aids in identifying key biomarkers that can be exploited for early diagnosis and non-invasive monitoring, advancing cancer care beyond late-stage treatment, and enabling precision oncology approaches [[7], [8], [9], [10], [11]]. In this review, we present an expanding overview of various tumor hallmarks in the framework of the cellular, intracellular, intercellular, and extracellular landscape for a better comprehension of the disease.

This review emphasizes the importance of structured segregation at cellular, intracellular, intercellular, and extracellular levels with detailed and incremental viewpoints on various hallmarks. This structured framework of tumor hallmarks may be helpful to better understand the complexity of cancer progression, how tumors evolve to drug resistance, and adaptation to new environments. In the translational perspectives, there is always a need to understand the tumor hallmarks for developing novel preventive, predictive, diagnostic, and prognostic avenues for the better management of cancer patients.

This review extends beyond traditional frameworks like Hanahan (2022) [8] in integrating existing and new hallmarks into a hierarchical model that encompasses intracellular, cellular, intercellular, and extracellular levels. This integrates systematically dimensions hitherto underrepresented within previous reviews, such as extrachromosomal circular DNA (eccDNA) [12], cross-organelle communication (e.g., crosstalk between mitochondria and ER) [13], immunometabolic regulation [14], and extracellular vesicle–mediated signaling [15]. We also highlight the merging of spatial and single-cell multi-omics capabilities to record tumor heterogeneity at previously unattainable resolution [16]. The originality of this consolidation is in converting these intricate layers into clinically relevant insights through connecting hallmarks with therapeutic targets, technologies, and evidence maps [17]. By offering systematic research questions and actionable entry points, for instance, how to validate biomarkers [18], how to stratify patients using multi-omics tools [19], and how to therapeutically target immune-stromal interactions [20]. The current framework, which builds on Hanahan (2022) [8], bridges conceptual gaps between intracellular mechanisms and intercellular dynamics by incorporating recently characterized molecular systems, including mitochondria-associated membranes (MAMs), eccDNA, and AI-enabled multi-omics integration. The connection between mechanistic biology and clinical innovation is strengthened when these elements are incorporated into a hierarchical structure, which permits a continuum between molecular regulation, cellular adaptation, and translational implementation. This review provides readers with a hands-on map connecting conceptual breakthroughs with translational oncology.

## 2 Hallmarks at the cellular level

Cancer is characterized by the continuous acquisition of diverse molecular, cellular, and spatial, including widely recognized hallmarks such as selective growth and proliferative advantage, apoptosis resistance with limitless replicative potential, invasion and metastasis, metabolic rewiring, supportive tumor microenvironment (TME), and immune modulation [[5], [6], [7], [8]]. In addition, the conceptual framework of cancer has evolved to incorporate emerging hallmarks such as immunological evasion, metabolic reprogramming, non-mutational epigenetic remodeling, polymorphic microbiome interactions, and phenotypic plasticity, which further empower cancer cells within the TME [9,10]. A chronological overview of the development of tumor hallmarks is presented (Table 1). Given the dynamic and evolving nature of these attributes, continued efforts to organize these various tumor hallmarks spanning cellular, intracellular, intercellular, and extracellular levels are essential for basic and translational contexts [11].

**Table 1:** Chronological overview of tumor hallmarks with notable altered genes and proteins. The table traces the historical progression of key hallmark concepts, from early discoveries such as apoptosis evasion and immortalization to recent hallmarks including epigenetic reprogramming and cancer stem cell maintenance. Representative genes/proteins and landmark references are provided.

Tumor Hallmark	Year	Notable Altered Genes/Proteins	Reference
Loss of Apoptosis	1990	TP53, BCL-2, BAX	41
Immortalization	1990	Telomerase (TERT), p16INK4A	30
Genetic Instability	1991	TP53, BRCA1, BRCA2	41, 43, 44
Uncontrolled Growth	2000	MYC, RAS, Cyclin D1	1
Evading Immune Surveillance	2000	CTLA-4, PD-1, PD-L1	1
Tumor-Promoting Inflammation	2000	NF-κB, COX-2, IL-6	1
Angiogenesis	2007	VEGFA, VEGFR, HIF-1α	47
Deregulated Metabolism	2009	GLUT1, HK2, PKM2	63
Genome Instability and Mutation	2009	APOBEC, PIK3CA, KRAS	40
Invasion and Metastasis	2020	MMPs, E-cadherin, N-cadherin	57
**Next Generation Tumor Hallmarks**			
Aberrant Cell Signaling	2011	RAS, RAF, MEK	3
Tumor Microenvironment Remodeling	2013	TGF-β, FGF, PDGF	78
Cancer Stem Cell Maintenance	2014	CD44, CD133, ALDH1	5,6
Epigenetic Reprogramming	2016	DNMT1, HDAC1, EZH2	86

### 2.1 Overexpression of cellular receptors

Receptor tyrosine kinases (RTKs), such as HER2 and EGFR, are central key drivers to achieve sustained oncogenic MAPK and PI3K/AKT signalling pathways [[3], [4], [5], [6], [7], [8], [9], [10], [11], [12], [13], [14]]. Mechanisms underlying receptor dysregulation include gene amplification, point mutations, aberrant splicing, and epigenetic modifications that enhance receptor stability and prevent degradation, leading to sustained signaling activity [21]. A highly studied receptor, HER2 amplification in breast cancer, enables ligand-independent dimerization with ErbB receptors, persistently activating downstream growth pathways and contributing to aggressive tumor behavior [22]. Similarly, in lung cancer, overexpression of CXCR7 facilitates tumor growth, angiogenesis, and metastasis by promoting cancer cell migration and establishing metastatic niches [23]. As our understanding deepens, it is increasingly evident that receptor dysregulation not only drives tumor progression but also shapes the TME and contributes to cancer heterogeneity [24].

Although emerging therapies, including bispecific antibodies, target these overexpressed receptors and have improved clinical outcomes, compensatory activation of alternative pathways and the development of resistance remain significant therapeutic challenges [[23], [24], [25], [26], [27]].

### 2.2 Enabling replicative immortality

In addition to receptor overexpression, another key cellular hallmark is enabling replicative immortality, a crucial acquired hallmark by cancer cells that allows them to surpass the process of cellular aging and the limit of cell division [20,28]. Replicative immortality, primarily driven by telomerase reactivation or alternative lengthening of telomeres (ALT) mechanisms, enables cancer cells to bypass normal cellular senescence. By maintaining telomere length and avoiding senescence, cancer cells gain the capacity for unlimited division, contributing to tumor growth and progression. This hallmark does not occur in isolation but is strongly associated with a range of genetic and epigenetic changes that contribute to the complex and heterogeneous nature of cancer [29,30].

When the telomeres become short, they activate the DNA damage response, leading to apoptosis or cell death. In most cancers, the telomerase enzyme is reactivated to maintain the length of the telomeres by adding repetitive nucleotides at the end of chromosomes. This prevents the telomeres from shortening and senescence in cancer [31]. In cancer, when the telomerase is not reactivated the cancer cells undergo an alternative mechanism called alternative lengthening of telomeres (ALT). It is a recombination-based process where the telomere length is maintained through homologous recombination [[32], [33], [34]]. ALT is most found in cancers such as osteosarcoma, glioblastoma, and certain soft tissue sarcomas.

Dual inhibitors or combination therapies targeting telomerase, along with DNA damage repair regulators such as ATR or PARP, offer avenues to prevent cancer cells from escaping. Reports on telomerase inhibitors, such as imetelstat, that can target replicative immortality, in myeloproliferative neoplasms and solid tumors are promising [[35], [36], [37]].

### 2.3 Genomic alterations

Genomic alterations due to mutations, rearrangements, and amplifications are known to drive tumor evolution. These changes, as diverse as the patients they inhabit, shape the hallmarks of cancer, creating a heterogeneous landscape where no two tumors are alike (3, 38). Cancer is emphasized for encompassing multiple genetic events, such as gain of function (GOF) of oncogenes and a loss of function (LOF) of tumor suppressor genes (TSG). The GOF mutations lead to the conversion of proto-oncogenes into oncogenes [38,39]. Notable examples of oncogenic GOF are discovered as copy number variations in HER2, point mutations in RAS (e.g., KRAS, HRAS, and NRAS), and chromosomal translocations of BCR-ABL fusion protein in CML [3].

Conversely, LOF mutation in TSG undergoes biallelic inactivation through point mutations, deletions, or epigenetic silencing, resulting in unchecked cell division, tumor progression, and evasion of cell death processes [40]. Some significant GOF of TSG genes are documented as TP53, RB1, and PTEN. The loss of TSG can also occur through haploinsufficiency, leading to modulation of the normal function of these TSGs [41]. For instance, in colorectal cancer, the inactivation of the APC tumor suppressor gene, activation of the KRAS oncogene, and subsequent loss of TP53 create a combined effect of functional gains and losses in achieving a pro-tumor genetic landscape [[42], [43], [44], [45]]. Additionally, in many cancers, a combination of multiple and sequential genetic mutations at individual cellular levels includes both oncogene GOF and TSG LOF to initiate tumorigenesis, perpetuation of infinite proliferation, development, invasion, and metastasis [[42], [43], [44]].

### 2.4 Transcriptional hallmarks

The genome can be viewed as a complex system where transcriptional hallmarks play a significant role in regulating the changes that drive tumor diversity and complexity [3]. Transcriptional dysregulation in cancer often involves changes in transcription factors, epigenetic regulators, chromatin modifiers, and other components of the transcriptional machinery [45]. Dysregulation of key oncogenes and tumor suppressor genes such as MYC, RAS, TP53, and RB1 can lead to uncontrolled cell growth and proliferation [46]. Transcription factors like NF-κB, STAT3, and HIF-1α frequently exhibit abnormal activity, supporting cancer progression through enhanced survival, inflammation, and angiogenesis. Aberrant epigenetic changes, including DNA methylation and histone modifications, can silence tumor suppressor genes or activate oncogenes, further driving malignancy [47]. Non-coding RNAs, such as miRNAs and lncRNAs, also contribute by regulating chromatin structure, mRNA stability, and transcription factor activity, promoting metastasis, therapy resistance, and cancer cell survival [48].

Additionally, alternative splicing and transcriptional plasticity create heterogeneity, allowing cancer cells to reprogram transcriptional networks to support tumor growth and adaptation. Advances in understanding transcriptional regulation have identified potential therapeutic targets such as super-enhancers, transcriptional addiction, and stress response pathways [49]. Transcriptional addiction by cancer is a preferred therapeutic target, and transcriptional inhibitors such as BRD4 inhibitors have shown promise in disrupting super-enhancer-driven transcription [[50], [51], [52]]. Combining transcriptional inhibitors with other forms of anticancer drugs, such as chemotherapeutic and immune checkpoint therapies, may offer a promising approach to improving treatment outcomes and preventing resistance [52].

### 2.5 Extrachromosomal circular DNAs

eccDNAs contribute to oncogene amplification, intratumoral heterogeneity, and therapy resistance, particularly through chromothripsis-driven genomic rearrangements [[53], [54], [55]]. These circular DNA elements are recognized for their role in driving key cancer hallmarks, including sustained proliferative signaling, evasion of growth suppressors, resistance to cell death, replicative immortality, angiogenesis, invasion, and metastasis [53]. A unique feature of eccDNAs is their ability to carry full-length oncogenes with high copy numbers and enhanced transcriptional activity, which contributes to aggressive tumor growth and progression [54].

EccDNAs arise from processes such as chromothripsis and DNA damage repair, and they exist in various forms, including telomeric circles (t-circles), small poly-dispersed DNA (spcDNA), microDNA, and larger eccDNAs of varied sizes. In glioblastoma, eccDNAs carrying EGFR drive sustained receptor tyrosine kinase signaling, while in lung cancer, eccDNAs amplify MYC and promote chromosomal instability and cell division [55]. eccDNAs carrying multiple copies of EGFR in glioblastoma and MYC in small-cell lung cancer have been shown to promote excessive proliferation and resistance to targeted therapies [53,56].

EccDNAs also contribute to metastasis by increasing the expression of genes associated with epithelial-mesenchymal transition (EMT) and matrix metalloproteinases (MMPs) [57,58]. Importantly, eccDNAs hold potential as non-invasive biomarkers for cancer detection and monitoring, as they can be identified in liquid biopsies, including blood and other bodily fluids, reflecting the mutational landscape of tumors [59].

eccDNA with oncogene amplifications like EGFR or MYC promotes intratumoral heterogeneity and resistance to therapy [60]. Its presence in the circulation provides a non-invasive window to observe tumor dynamics [61]. eccDNA also has clinical promise as a diagnostic biomarker, where longitudinal profiling can monitor clonal evolution in response to therapy. eccDNA-initiated amplification events can also foresee resistance to kinase inhibitors, making eccDNA a candidate for monitoring resistance and an adjunct to traditional genomic sequencing for companion diagnostics [62].

### 2.6 Deregulating cellular energetics and metabolism

By contrast to genomic instability, which alters DNA structure, cancer cells also undergo significant metabolic reprogramming to support rapid growth, survival, and adaptation to hostile environments. Deregulated metabolism is now recognized as a core hallmark of cancer, essential for meeting the biosynthetic, bioenergetic, and redox requirements of uncontrolled proliferation [3]. One of the best-known metabolic shifts is the Warburg effect, where cancer cells preferentially use aerobic glycolysis, converting glucose to lactate even in the presence of sufficient oxygen, which supports proliferation despite lower energy efficiency [63]. Although glycolysis is upregulated, mitochondrial function remains active, and cancer cells can switch to mitochondrial respiration when glucose is limited.

Lipid metabolism is also altered to support membrane synthesis, energy storage, and signaling, often through increased fatty acid oxidation, depending on the cancer type. Key regulators of lipid metabolism include Sterol Regulatory Element-Binding Proteins (SREBPs), Fatty Acid Synthase (FASN), and AMP-Activated Protein Kinase (AMPK) [64,65]. Additionally, glutamine metabolism is reprogrammed in many cancers to support nucleotide synthesis and maintain redox balance. Hypoxia and mutations in p53 further contribute to metabolic changes, enabling cancer cells to survive under stress and promoting immune evasion by limiting immune cell infiltration and enhancing the expression of immune checkpoint molecules [9,66].

Therapeutic strategies under investigation include glycolysis inhibitors (e.g., 2-deoxy-d-glucose), lipid metabolism inhibitors (e.g., FASN inhibitors), and agents targeting mitochondrial function. The emerging field of immunometabolism explores how metabolic changes in the TME suppress immune responses and how metabolic inhibitors combined with immune checkpoint blockade may enhance anti-tumor immunity [67,68].

### 2.7 Resisting cell death

Resisting cell death is a critical survival strategy for cancer cells, enabling uncontrolled proliferation and contributing to therapy resistance (3). This hallmark poses a significant challenge in cancer treatment. In normal cells, programmed cell death (apoptosis) is tightly regulated by intrinsic and extrinsic pathways, primarily controlled by the BCL-2 family of proteins, which balance pro-apoptotic factors (such as BAX and BAK) and anti-apoptotic factors (such as BCL-2 and BCL-XL) [69]. The intrinsic pathway is governed by mitochondrial outer membrane permeabilization (MOMP), which regulates the release of cytochrome *c* from mitochondria [70]. Upon release, cytochrome *c* binds to Apaf-1, forming the apoptosome that activates caspase-9, leading to the activation of downstream caspases and cell death. In cancer cells, this process is inhibited through overexpression of anti-apoptotic proteins such as BCL-2, MCL-1, and BCL-XL, preventing cytochrome *c* release. The extrinsic pathway is triggered by ligands like FasL and TRAIL binding to death receptors on the cell surface, forming a death-inducing signaling complex (DISC) and activating caspase-8 [71].

Cancer cells often evade this pathway by overexpressing FLIP or reducing the expression of death receptors like Fas. Resistance to apoptosis has become a key therapeutic target, leading to the development of agents such as BCL-2 inhibitors, TRAIL receptor agonists, and SMAC mimetics, although resistance to these therapies can still emerge. Beyond apoptosis, cancer cells can evade other forms of programmed cell death, including necroptosis, pyroptosis, and ferroptosis [72,73]. Recently, combining apoptosis-inducing agents with metabolic inhibitors or immune checkpoint blockers are being investigated to overcome resistance [74,75].

### 2.8 Epigenetic reprogramming

Epigenetic reprogramming, a reversible and dynamic process, is now recognized as a hallmark of cancer. Unlike genetic alterations, which change the DNA sequence, epigenetic changes modify gene expression without altering the underlying base pairs. These changes play a key role in the tumor initiation, growth, angiogenesis, and resistance to growth inhibition, allowing cancer cells to survive in hostile environments [47,[76], [77], [78], [79], [80]].

Epigenetic reprogramming involves modifications such as DNA methylation, histone alterations, and chromatin remodeling, which collectively regulate gene expression. Aberrant epigenetic changes can activate oncogenes or silence tumor suppressor genes, contributing to malignant transformation [76]. Hypermethylation of promoter regions in genes like p16 and BRCA1 leads to their silencing, while hypomethylation of repetitive DNA elements destabilizes the genome. Histone post-translational modifications, including acetylation and methylation, influence chromatin accessibility, and transcriptional activity. Non-coding RNAs, such as microRNAs and long non-coding RNAs, further regulate gene expression by modulating mRNA stability and translation [48].

DNA methylation-mediated silencing of tumor suppressor genes is known to enable cancer cells to bypass growth inhibition. Histone modifications may activate oncogenic pathways like Wnt/β-catenin and NF-κB [77,78]. Key mechanisms of epigenetic regulation include DNA methylation by DNMTs, histone modifications by HDACs and histone methyltransferases, and chromatin remodeling by complexes like SWI/SNF [[80], [81], [82]]. Epigenetic reprogramming is also linked to other hallmarks, including immune evasion and metabolic reprogramming, by regulating genes involved in immune checkpoints and metabolic enzymes (80–82).

**Fig. 1:**
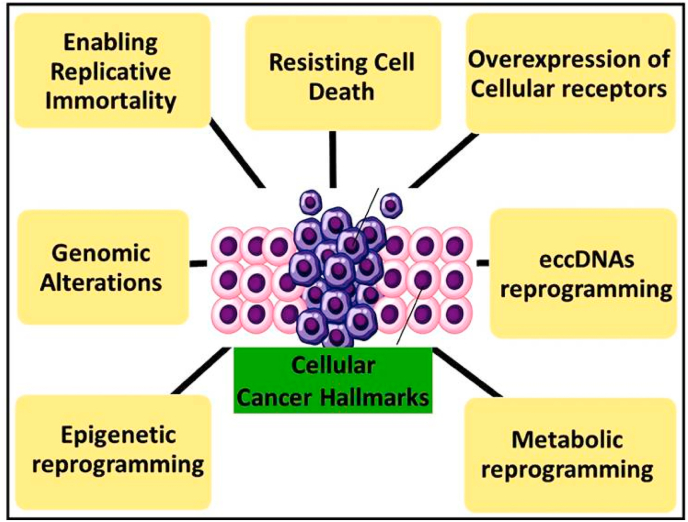
Summary of cellular cancer hallmarks. Key features at the cellular level are illustrated, including uncontrolled growth and proliferation, resistance to programmed cell death, and metabolic as well as epigenetic reprogramming. These alterations form the foundation of malignant transformation and sustained tumor growth.

Drugs such as DNA methyltransferase (DNMT) inhibitors and histone deacetylase (HDAC) inhibitors have shown promise in reactivating silenced tumor suppressor genes and restoring normal gene expression (79). Epigenetic therapies, such as DNMT inhibitors (e.g., azacitidine) and HDAC inhibitors (e.g., vorinostat), are already in clinical use, with more drugs under development (83). Understanding epigenetic reprogramming provides new insights into cancer biology and potential therapeutic targets (84–86). A summary of the cellular hallmarks of cancer is provided in Fig. 1, and key genes with their preclinical and clinical implications are detailed in Table 2.

**Table 2:** Summary of cellular hallmarks of tumors. Cellular-level mechanisms such as sustained proliferation, growth suppression evasion, replicative immortality, and deregulated energetics are described along with their associated genes, preclinical implications, and clinical relevance. The table highlights how these alterations provide therapeutic opportunities, such as EGFR and BCL2 inhibitors.

Cellular Tumor Hallmarks	Mechanism	Key Genes	Preclinical Implications	Clinical Implications	Reference
**Sustained Proliferation**	Hyperactivation of growth factor signaling	EGFR, KRAS, PIK3CA	Enhanced tumor growth, invasion	Target for cancer therapy (e.g., EGFR inhibitors)	[3,21]
**Evading Growth Suppression**	Inactivation of tumor suppressor genes	TP53, RB1, PTEN	Reduced apoptosis, increased proliferation	Poor prognosis, therapy resistance	[38,39]
**Enabling Replicative Immortality**	Telomere maintenance, ALT pathway	TERT, POT1, RAP1	Unlimited cell divisions	Cancer stem cell maintenance	[29,31]
**Resisting Cell Death**	Apoptosis evasion, anti-apoptotic protein expression	BCL2, BCLXL, MCL1	Reduced chemotherapy efficacy	Target for cancer therapy (e.g., BCL2 inhibitors)	[3,74]
**Deregulating Cellular Energetics**	Enhanced glycolysis, glutaminolysis	HK2, PKM2, GLS1	Metabolic reprogramming	Target for cancer therapy (e.g., HK2 inhibitors)	[63,83]
**Evading Immune Destruction**	Immune checkpoint regulation, immune suppression	CTLA4, PD1, PDL1	Reduced anti-tumor immunity	Immunotherapy (e.g., checkpoint inhibitors)	[26,84]
**Epithelial-to-Mesenchymal Transition (EMT)**	Loss of epithelial markers, gain of mesenchymal markers	SNAIL, TWIST, ZEB1	Enhanced migration, invasion	Poor prognosis, metastasis	[85,86]

Taken together, these cellular mechanisms form the foundation for deeper intracellular alterations that sustain tumor heterogeneity.

## 3 Hallmarks at the intracellular level

To achieve clarity and reproducibility, we systematically classified emerging tumor hallmarks using a rule-based approach instead of narrative intuition (8). The major criterion is the biological action site: events limited to organelles or genomes are intracellular; whole-cell-level processes (e.g., proliferation, immune evasion) are cellular; junction-mediated, cytokine or vesicle-mediated cross-talk between cells is intercellular; and signals transduced into or operating through extracellular fluids and matrices are extracellular (87). As a second rule, we further factored in direction of signal flow —that is, whether the hallmark arises within the cell and extends outward (e.g., EV release), or is externally driven and works inward (e.g., cytokine-induced signalling) (88). This two-rule classification enables systematic placement of markers into hierarchical tiers and adds to the framework's reproducibility.

### 3.1 Abnormal intracellular transport

Abnormal intracellular transport, including dysregulated vesicle trafficking and impaired cytoskeletal motor functions, is emerging as a potential hallmark of cancer due to its pivotal role in cell polarity, proliferation, invasion, immune evasion, and therapeutic resistance. Experimental studies show that mutations and overexpression of vesicular trafficking regulators such as Rab GTPases are intricately linked to tumor progression. For example, gain-of-function mutations in Rab35 promote the recycling of PDGFRα, leading to constitutive activation of PI3K/AKT signaling in the absence of ligands [85]. Similarly, overexpression of Rab23 and Rab25 enhances cancer cell invasion and metastasis by activating NF-κB and PI3K/AKT pathways [85,86]. Disrupted vesicle trafficking impairs epithelial cell polarity and contributes to early carcinogenic processes, including metastasis initiation [87,88].

Motor proteins essential for intracellular transport also influence cancer progression. Inhibition of kinesin Eg5 by agents like Dimethylenastron reduces tumor cell migration and invasion in pancreatic and breast cancer models [89]. Dynein inhibition by Dynarrestin impairs cytokinesis, proliferation, and c-Met signaling [90]. Non-muscle myosin II inhibitors, such as blebbistatin, significantly suppress migration and proliferation in pancreatic adenocarcinoma [91]. Microtubule-targeting agents, including paclitaxel and docetaxel, disrupt intracellular transport, inhibit mitosis, and suppress metastasis [92]. Cytoskeletal dynamics are further linked to autophagy modulation, immune evasion, and therapy resistance [[87], [88], [89], [90], [91], [92]]. These findings support the inclusion of abnormal intracellular transport as a cancer hallmark and present new opportunities for therapeutic intervention.

### 3.2 Intracellular altered metabolic-epigenomic profiles

The modified intracellular metabolic and epigenomic profiles of cancer cells reflect unique acquired capabilities of cancer cells. These combined molecular programs enable tumors to proliferate, adapt rapidly, and develop resistance throughout evolution, contributing to the well-documented heterogeneity of cancer. Decoding these metabolic-epigenomic changes advances our understanding of how the crosstalk between metabolism and epigenetics drives incremental cancer hallmarks, supporting cancer cell survival, adaptation, and resistance [[92], [93], [94], [95]].

Recent studies have shown that chromatin accessibility is strongly linked to cancer development. Chromatin accessibility, a marker of active regulatory DNA elements, reflects transcription factor binding within gene promoter regions. However, studies exploring the relationship between chromatin accessibility, tumor-infiltrating immune cells, and differentially expressed genes remain limited [92]. ATAC-seq is a powerful technique used to map open chromatin regions, nucleosome positioning, and transcription factor binding. Analysis of TCGA ATAC-seq datasets has identified genes with accessible promoter regions that are potentially regulated by specific transcription factors [93].

Chromatin function in cancer involves complex interactions between chromatin-modifying enzymes. Alterations in histone lysine methylation pathways are frequently observed in tumors, and specific chromatin mutations, such as the histone H3.3 Lys27-to-methionine substitution in juvenile glioma, exhibit disease-specific patterns [94]. Chromatin structure plays a critical role in gene expression regulation. Cancer cells disrupt the epigenomic architecture of healthy cells, where both genetic mutations and epimutations contribute to tumor initiation and progression [95]. Global DNA methylation changes, histone modification abnormalities, and dysregulation of chromatin-modifying enzymes lead to widespread gene expression alterations that promote cancer development [96].

Traditional oncogene activation models involving mutations, gene amplification, and chromosomal rearrangements are insufficient to fully explain oncogenic activation [97]. Transposable elements (TEs), normally epigenetically silenced, can become active in cancer due to DNA hypomethylation, exposing regulatory sequences with functional consequences [98]. TEs are abundant sources of regulatory elements. Inflammation and carcinogen exposure can upregulate TEs in precancerous cells, enhancing epigenetic plasticity and reprogramming transcriptional networks to support survival, genome instability, oncogene activation, and tumor suppressor inhibition [99]. TE dysregulation contributes to gene expression variability, genome instability, and cancer evolution, influenced by age, environment, and lifestyle factors [100].

Clayton et al. (2020) demonstrated that TE-derived alternative splicing is linked to gene fusion events in cancer, particularly involving the KLK2 gene. They proposed that TE sequence-mediated *trans*-splicing, potentially facilitated by heterogeneous ribonucleoprotein particles (hnRNPs), could underlie these fusion transcripts [101,102]. LINE1 and other TEs regulate genome diversity and chromatin dynamics, suggesting novel roles in cancer biology and therapeutic resistance [47].

Aberrant DNA methylation patterns significantly influence drug sensitivity and gene expression. Generally, hypermethylation of promoter CpG islands silences tumor suppressor genes, while hypomethylation activates oncogenes [103,104]. Hypoxia is another key driver in tumor biology. Due to insufficient vascularization relative to proliferation rates, tumors often develop hypoxic regions, shifting metabolism toward anaerobic glycolysis [105]. Interestingly, even in normoxia, cancer cells often maintain aerobic glycolysis, producing excess lactate.

HIF-1α, a central hypoxia-responsive transcription factor, stabilizes under low oxygen by escaping proteasomal degradation due to reduced prolyl hydroxylase (PHD) activity. Lactate accumulation can inhibit PHD2, even in oxygen-rich conditions, leading to “pseudohypoxia” where HIF-1α remains stabilized despite normoxia [106]. This condition enables cancer cells to mimic hypoxic responses, promoting survival and metabolic reprogramming [63].

Highly proliferative tumors face both hypoxia and nutrient scarcity, triggering adaptive responses to minimize energy consumption and activate alternative metabolic pathways [107]. These adaptations also promote migratory behaviors, including epithelial-mesenchymal transition (EMT), mesenchymal-to-amoeboid transition, and collective-to-amoeboid transition [79]. Under severe metabolic stress or experimentally induced HIF signaling, cancer cells may switch to amoeboid movement, characterized by reduced adhesion and bleb-based migration. This transition is driven by calpain-2-mediated cleavage of talin, weakening cell-ECM adhesion [108]. These bioenergetic and migratory adaptations further support cancer progression and metastasis.

### 3.3 Inter-organelles crosstalk

Organelle communication is a critical but often underappreciated mechanism that cancer cells exploit to support growth, survival, and resistance. One of the key examples is the interaction between the endoplasmic reticulum (ER) and mitochondria at membrane contact sites known as mitochondria-associated membranes (MAMs), which regulate calcium flux to balance cell survival and apoptosis. In glioblastoma, hyperactive MAMs enhance resistance to apoptosis by supporting mitochondrial energy production. In pancreatic cancer, lysosomal stress pathways enable cancer cells to survive nutrient deprivation. Targeting these organelle hubs, such as through MAM disruptors or autophagy inhibitors, may impair the adaptability of cancer cells [3].

As cancer cells progress from normal to malignant states, they acquire functional traits that depend on complex organelle interactions. Increasing evidence highlights inter-organelle communication as a key regulator of cancer cell behavior, linking metabolic adaptation, stress responses, and survival signaling [3].

The major organelles involved include mitochondria, ER, lysosomes, and peroxisomes. Mitochondria and ER are intricately connected via MAMs, which regulate calcium signaling and lipid exchange. This interaction is essential for maintaining mitochondrial energy production and can regulate apoptosis, particularly under oncogenic stress [109]. In many cancers, increased ER-mitochondrial coupling promotes calcium transfer that enhances mitochondrial metabolism, supporting tumor growth. In glioblastoma, elevated calcium signaling at MAMs drives ATP production and contributes to apoptosis resistance.

Lysosome-mitochondria interactions also play critical roles in cancer adaptation. Lysosomes, central to autophagy, degrade and recycle damaged cellular components, providing essential metabolites to fuel mitochondrial function (114). This process, known as mitophagy, maintains mitochondrial health. In hepatocellular carcinoma, mitophagy is elevated under hypoxic conditions to preserve mitochondrial integrity and prevent cell death. Lysosomes also influence mitochondrial activity through signaling molecules such as reactive oxygen species (ROS), which can stabilize hypoxia-inducible factor 1-alpha (HIF-1α) and support cancer cell survival under low oxygen conditions. (115)

Peroxisome-mitochondria communication is another important axis in cancer. Peroxisomes contribute to lipid metabolism and produce hydrogen peroxide (H2O2), which serves as a signaling molecule in cancer. In breast cancer, these interactions enhance fatty acid oxidation to support energy demands, while increased H2O2 signaling promotes cell proliferation and migration via PI3K/AKT and MAPK pathways [110].

ER-lysosome communication is essential for cancer cells to adapt to nutrient stress. Lysosomes break down cellular components to release amino acids, which are used by the ER for protein synthesis, allowing cancer cells to survive during nutrient deprivation. In ovarian cancer, ER-lysosome coupling sustains autophagy to maintain tumor growth even in nutrient-poor environments [111].

Nuclear-mitochondrial cross-talk also contributes to cancer progression. Mitochondrial dysfunction can trigger retrograde signaling to the nucleus, reprogramming gene expression to favor glycolysis over mitochondrial respiration, as observed in colorectal cancer. This supports the Warburg effect and enhances cancer cell survival [112]. Mitochondrial oxidative stress can also activate nuclear DNA repair pathways, contributing to genomic stability and resistance to cell death.

Together, these organelle interactions enable cancer cells to adapt to hostile conditions, evade apoptosis, and meet their metabolic demands. Disrupting these inter-organelle communication pathways offers a promising strategy to limit cancer cell adaptability and growth. Further mechanistic studies are essential to identify new therapeutic targets within these organelle networks [113,114].

ER–mitochondria coupling controls calcium flow and metabolic reconfiguration, facilitating apoptotic evasion and therapy resistance [115]. At the clinic, the new hallmark implies that inhibiting calcium transfer proteins or tethering factors at mitochondria–ER junctions might resensitize tumors to pro-apoptotic drugs [116]. Tracing organelle contact signatures would yield biomarkers of cellular stress and adaptation, and MAM protein expression detection could be a companion diagnostic in apoptosis-targeted therapeutic trials [117]. Furthermore, inter-organelle communication profoundly influences metabolic fluxes, linking structural alterations with the metabolic phenotype described below.

### 3.4 Warburg effects

Cancer cells undergo metabolic reprogramming, most prominently the Warburg effect, wherein they preferentially use aerobic glycolysis over oxidative phosphorylation even in oxygen-rich conditions [3,9]. This shift, although less efficient for ATP production, supports the biosynthetic and metabolic demands of rapidly proliferating tumor cells, providing precursors for nucleotide, lipid, and amino acid synthesis and regulating lipid homeostasis [118]. HIF-1α stabilization under hypoxia enhances glycolytic flux, while lactate accumulation generates a “pseudohypoxic” microenvironment that promotes tumor progression. In neuroblastoma, inhibition of LDHA suppresses proliferation, highlighting the therapeutic potential of targeting glycolysis [119].

Key regulators of this metabolic phenotype include glucose transporters GLUT1/GLUT3, mitochondrial pyruvate carriers MPC1/2, PDH inactivation via PDK1/PDK3, and oncogenic signaling through EGFR and mutant K-Ras [[119], [120], [121]]. Cancer cells compensate for glycolysis's low ATP yield via accelerated flux and glutamine metabolism, with feedback on PFK-1 overridden by elevated fructose-2,6-bisphosphate through PFKFB3/4, while downregulation of TIGAR in p53-mutant tumors sustains glycolysis [110,122].

This metabolic rewiring directly affects immune metabolism. High glucose consumption and lactate accumulation suppress effector T-cell activity, NK cell function, and dendritic cell activation, fostering an immunosuppressive tumor niche. Correlations between Warburg-associated proteins and immune infiltration underscore these effects: GLUT1 and LDH5 inversely correlate with CD8+/CD3+ T cells in renal cell carcinoma, MCT4 enhances dendritic and macrophage presence but reduces B cell infiltration in breast cancer, and PKM2 dampens CD8^+^ T cell function [123,124]. Detailed mechanisms linking tumor metabolism to immune modulation are discussed in Section 2.2.

**Fig. 2:**
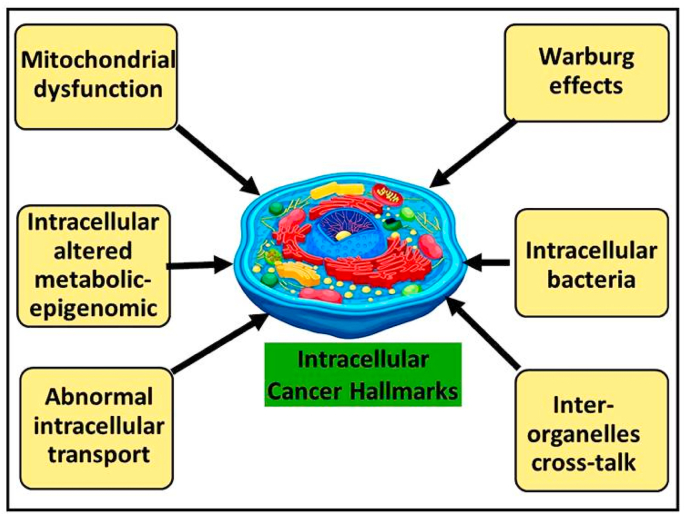
Model of intracellular tumor hallmarks. The figure highlights mitochondrial dysfunction, disrupted intracellular transport, and dysregulated signalling cascades that collectively reprogram cancer cell metabolism, promote stress adaptation, and enhance survival.

Beyond classical glycolysis, integrating metabolic, immunologic, and epigenetic dimensions provides deeper explanatory power. The immunometabolic–metabolism–epigenetic–immune axis illustrates how metabolic rewiring drives epigenetic plasticity, immune evasion, and therapy resistance, while inter-organelle crosstalk among mitochondria, ER, and lysosomes reveals novel mechanisms of drug tolerance [[125], [126], [127]]. These interconnected “closed loops” do not replace classical hallmarks but elaborate on them, offering testable hypotheses for unresolved clinical challenges. Mechanistic details and pathways are summarized (Fig. 2 and Table 3).

**Table 3:** Summary of intracellular tumor hallmarks. Key intracellular mechanisms including genomic instability, epigenetic alterations, mitochondrial dysfunction, altered cell cycle regulation, aberrant signaling, impaired apoptosis, and autophagy dysregulation are listed with representative genes. Preclinical and clinical implications emphasize therapeutic strategies such as PARP inhibitors, HDAC inhibitors, and autophagy modulators.

Intracellular Tumor Hallmarks	Mechanism	Key Genes	Preclinical Implications	Clinical Implications	Reference
**Genomic Instability**	Mutations, chromosomal instability	TP53, BRCA1, BRCA2	Enhanced tumorigenesis	Target for therapy (e.g., PARP inhibitors)	[98,99]
**Epigenetic Alterations**	DNA methylation, histone modification	DNMT1, HDAC1, EZH2	Silencing tumor suppressors	Target for therapy (e.g., HDAC inhibitors)	[79,102]
**Mitochondrial Dysfunction**	Altered metabolism, ROS production	SDHB, SDHD, VDAC2	Enhanced tumorigenesis	Target for therapy (e.g., metformin)	[108,121]
**Altered Cell Cycle Regulation**	Hyperactivation of CDKs, inactivation of CKIs	CDK4, CDK6, p16	Uncontrolled proliferation	Target for therapy (e.g., CDK4/6 inhibitors)	[38,39]
**Aberrant Signaling Pathways**	Activation of RTKs, RAS/MAPK pathway	EGFR, KRAS, BRAF	Enhanced tumorigenesis	Target for therapy (e.g., EGFR inhibitors)	[44,45]
**Impaired Apoptosis**	Overexpression of anti-apoptotic proteins	BCL2, BCLXL, MCL1	Reduced chemotherapy efficacy	Target for therapy (e.g., BCL2 inhibitors)	[119,128]
**Autophagy Dysregulation**	Altered autophagy flux, lysosomal dysfunction	ATG5, ATG7, LAMP2	Enhanced tumorigenesis	Target for therapy (e.g., autophagy inhibitors)	[129,130]

## 4 Hallmarks at intercellular level

### 4.1 Intercellular signaling network

Inter-organelle crosstalk within cells is essential for maintaining coordinated cellular functions. This complex communication network is often disrupted in cancer, contributing to tumor plasticity, adaptability, and progression [3,9]. Understanding these communication pathways provides insight into the survival strategies of cancer cells and may reveal novel therapeutic targets.

Communication between pre-neoplastic or malignant cells and surrounding stromal, immune, and host cells plays a critical role in tumor initiation and progression [3]. Gap junctional intercellular communication (GJIC) has long been implicated in both cancer development and progression and may present a future therapeutic target. Gap junctions (GJs) are specialized structures that connect the cytoplasms of adjacent cells, facilitating the passive diffusion of small molecules.

Tight junctions (TJs) also play a crucial role, particularly in the preliminary stages of metastasis. Loss of TJs disrupts cell polarity, adhesion, differentiation, and motility, promoting tumor cell invasion [131]. Additional intercellular junctions, such as adherens junctions (AJs) and desmosomes (DSMs), are also essential in epithelial tissues. Both rely on cadherins and related adhesion proteins but differ from TJs by connecting to cytoskeletal components rather than forming membrane seals.

The temporal and functional dynamics of these junctions during tumor progression vary. In vivo studies indicate that DSMs are lost earlier than AJs during carcinogenesis and early invasion. Loss of DSMs weakens cell adhesion, increases local invasion, promotes cell survival, and allows infiltration of inflammatory cells. Subsequent AJ loss further enhances cellular invasion and distant metastasis [132].

Exosomal vesicles (EVs) have emerged as key mediators of intercellular communication in cancer. EVs can transfer oncogenic molecules between cancer cells and other cell types in the TME, including stromal and immune cells [133]. They facilitate both local and long-distance communication, including interactions between primary tumors and metastatic sites. EVs contribute to local invasion, epithelial-mesenchymal transition (EMT), extracellular matrix (ECM) remodeling, angiogenesis, and various stages of metastasis [134].

Recent discoveries have also identified tunneling nanotubes (TNTs) as important structures in cancer cell communication. TNTs are dynamic, membrane-bound channels ranging from 50 to 800 nm in diameter that allow the transfer of diverse cargo, including proteins, calcium ions, nucleic acids, non-coding RNAs, and even entire organelles such as mitochondria, lysosomes, and autophagosomes. TNT-mediated communication occurs between cancer cells and between cancer and stromal cells, facilitating cellular adaptation and survival under metabolic and environmental stress [135].

Together, these interconnected communication pathways, gap junctions, tight and adherens junctions, desmosomes, EVs, and TNTs—play critical roles in tumor development, progression, metastasis, and therapy resistance [136].

TNTs and EVs facilitate the exchange of mitochondria, proteins, and miRNAs between tumor cells, maintaining resistant subpopulations following treatment [137]. In translation, EV cargo like miRNA-21 or PD-L1 holds promise as liquid biopsy biomarkers, and TNT-mediated mitochondrial transfer as a therapy adaptation marker [138]. These processes not only account for drug resistance but also reveal potential areas for intervention, e.g., inhibiting TNT formation or altering EV release and cargo. These approaches could potentially stratify patients to receive immunotherapy and enhance monitoring of response to treatment [139].

### 4.2 Exosomal vesicles mediated intercellular exchange

EVs are key mediators of intercellular communication, facilitating the transfer of proteins, lipids, and nucleic acids between cells. In cancer, EVs play a crucial role in supporting tumor growth, metastasis, immune evasion, and resistance to therapy [87]. Within the TME, complex interactions occur between tumor cells and surrounding non-cancerous cells, including fibroblasts and macrophages. These interactions involve both paracrine signaling via growth factors and cytokines, and direct cell-to-cell contact. Tumor cells secrete factors such as PDGF, CSF1, and VEGF that recruit macrophages to the tumor site [136].

EVs are a heterogeneous population of membrane-bound vesicles secreted by various cell types. They encapsulate bioactive molecules within a lipid bilayer, protecting their contents from enzymatic degradation. Tumor-derived EVs specifically influence the TME by transferring oncogenic proteins, nucleic acids, and signaling molecules to stromal and immune cells, promoting tumor growth, invasion, and metastasis [140].

The formation of EVs involves the inward budding of the limiting membrane of endosomes, leading to the development of multivesicular endosomes (MVEs). These MVEs either fuse with lysosomes for degradation or with the plasma membrane to release their intraluminal vesicles as exosomes into the extracellular space [141].

TDEs contribute to the reprogramming of stromal cells, particularly by inducing the differentiation of fibroblasts into cancer-associated fibroblasts (CAFs), which create a stroma supportive of tumor progression [141]. Additionally, TDEs can promote pathological angiogenesis by delivering angiogenic factors to endothelial cells, facilitating the formation of new blood vessels that sustain tumor growth.

EVs also modulate immune responses within the TME. For example, TDEs can carry Fas ligand (Fas-L), which interacts with Fas receptors on immune cells to induce apoptosis. This mechanism suppresses anti-tumor immunity by promoting the death of CD8^+^ cytotoxic T cells and enhancing the proliferation of regulatory T cells, aiding immune evasion [142].

The TME exhibits significant immune heterogeneity, with tumor cell subpopulations dynamically interacting with immune cells and stromal elements in both cooperative and competitive ways. Single-cell sequencing has shown that a wide variety of immune cells, including macrophages, NK cells, mast cells, innate leukocytes, B cells, T cells, and memory cells, infiltrate tumors [143].

This heterogeneity presents a major challenge to effective immunotherapy. Differences in immune cell infiltration between primary tumors and metastases, or among metastases within the same patient, reflect subclonal variability. Studies analyzing CD8^+^ T cell distribution in melanoma samples revealed significant variability in immune cell presence, even within different regions of the same tumor [144,145].

Various immune cell subsets, including myeloid antigen-presenting cells [144], CAFs, and cytotoxic T lymphocytes (CTLs) [145], infiltrate tumors in a highly heterogeneous and spatially variable manner. These heterotypic interactions profoundly shape tumor progression and influence therapeutic outcomes.

### 4.3 Activation of cancer-associated fibroblasts

CAFs are key facilitators of tumor progression. They secrete extracellular matrix (ECM)-remodeling enzymes, such as matrix metalloproteinases (MMPs) and lysyl oxidase (LOX), which create pathways for tumor invasion. CAFs also release exosomes, including TGF-β–laden vesicles, which contribute to metastatic niche formation. Their plasticity, often driven by tumor-derived IL-6, makes them adaptable and resistant to therapy. CAF activation is triggered by a range of signals from cancer cells and the surrounding stroma and is closely linked to tumor heterogeneity and complexity [86]. Understanding CAF activation is essential for advancing targeted cancer therapies.

The TME is a complex and heterogeneous system composed of tumor cells, immune cells, stromal cells, and vascular components, all of which shape tumor development [146]. Fibroblasts are the most abundant stromal cells and contribute to the ECM. They are activated by inflammatory cytokines produced by tumor cells, immune cells, and stromal cells within the TME. These activated fibroblasts, termed CAFs, can also arise from epithelial cells, blood vessels, adipocytes, pericytes, and smooth muscle cells through epithelial-mesenchymal transition (EMT) or endothelial-mesenchymal transition (EndMT) [147].

CAFs regulate the tumor stroma and are involved in immunosuppression, angiogenesis, ECM remodeling, and the maintenance of cancer stem cells, contributing to therapy resistance. CAFs are not genetically mutated but express mesenchymal markers such as vimentin, α-SMA, FAP, and PDGFR-α, while lacking epithelial, endothelial, or hematopoietic markers. They display enhanced secretory, migratory, and proliferative properties [148] and produce ECM components like tenascin, periostin (POSTN), and secreted protein acidic rich in cysteine (SPARC). Pancreatic stellate cells, normally quiescent fibroblasts in the liver and pancreas, can be activated into CAFs by PDGF and TGF-β. TGF-β is the most abundant CAF-secreted factor and a key regulator of fibrosis and tumor-stroma interactions [149].

CAF activation is driven by oxidative stress, tumor-derived growth factors, and hypoxic conditions. Critical activators include TGF-β, EGF, FGF2, and PDGF. IL-6 secreted by CAFs activates the JAK/STAT3 pathway in tumor cells, promoting proliferation via cyclin D1 upregulation. While conventional chemotherapy targets dividing cells, it often fails to eliminate CAFs, some of which can adopt aggressive phenotypes and resist apoptosis, even entering senescence [150].

Activated CAFs reorganize the extracellular matrix and produce cytokines such as TGF-β, IL-6, and CXCL12, which reinforce immune suppression and therapy resistance [151] CAF-derived markers like α-SMA or FAP can help in diagnosis and patient stratification, while CAF signatures can act as prognosticators of immunotherapy [152]. Through the connection of CAF biology with drug resistance, this aspect proposes therapeutic interventions based on CAF reprogramming or CAF-secreted cytokine targeting to improve checkpoint blockade effectiveness [153].

### 4.4 Immune cell evasion

In addition to fibroblast-driven remodeling of the tumor stroma, immune modulation emerges as another cooperative mechanism that sustains tumor growth.

Immune evasion is a crucial survival strategy for tumors. It involves immune checkpoint upregulation (e.g., PD-L1), manipulation of myeloid cells (e.g., TAMs and MDSCs), and antigen presentation impairment (e.g., reduced MHC expression). These mechanisms contribute to immune resistance and the development of ‘cold’ tumors, which are less responsive to immunotherapy [3,26,154].

Tumor-infiltrating immune cells (TIICs) may initially exert anti-tumor effects but can be reprogrammed by tumor-derived factors to support tumor growth. Tumors commonly show low MHC expression and downregulated antigen-processing machinery (LMP2, LMP7, TAP, tapasin), reducing immune recognition. Tumor-associated antigens (TAAs) can stimulate T and NK cells, but their low immunogenicity, partly because some TAAs are self-antigens, limits effective immune responses [155,156].

Cytokines play a central role in shaping the tumor immune microenvironment. Interleukin-6 (IL-6) activates the JAK/STAT3 pathway, promoting tumor cell proliferation and immune suppression. Tumor necrosis factor-α (TNF-α) enhances epithelial–mesenchymal transition (EMT) and facilitates metastasis, while transforming growth factor-β (TGF-β) drives fibroblast activation, immunosuppressive regulatory T cell expansion, and reduced cytotoxic T cell activity [[157], [158], [159]]. Together, these cytokines remodel the tumor milieu into an immunosuppressive niche that diminishes the efficacy of immune checkpoint inhibitors and accelerates tumor progression [160].

Immune checkpoint proteins (ICPs) are often overexpressed in tumors, leading to suppressed immune activity. ICPs also contribute to tumor survival, metastasis, and therapy resistance [161]. Cancer cells reprogram metabolism to meet their energy demands, prominently exhibiting the Warburg effect, enhanced aerobic glycolysis. In hepatocellular carcinoma, the CD147-PI3K/Akt/mTOR axis drives the Warburg effect and suppresses T cell function [162]. In non-small cell lung cancer, PD-L1 promotes the Warburg effect by activating HK2, reducing CD8^+^ T cell activity.

Peripheral blood monocytes (CD14hi CD16lo and CD14lo CD16hi) are recruited into tumors and differentiate into tumor-associated macrophages (TAMs). TAMs polarize into M1 (anti-tumor) or M2 (pro-tumor) phenotypes, with M2 macrophages being predominant in tumors. TAMs promote angiogenesis, EMT, ECM remodeling, immune suppression, and cancer cell proliferation [163,164]. They express PD-L1/PD-L2 and CD80/CD86, which interact with PD-1 and CTLA4 on T cells to inhibit anti-tumor responses.

Tumor-associated neutrophils (TANs) release cytokines, chemokines, and enzymes like MMPs and neutrophil elastase, contributing to tumor invasion and metastasis [165]. Myeloid-derived suppressor cells (MDSCs), expanded by tumor-derived signals, produce immunosuppressive cytokines (e.g., TGF-β, IL-10) and enzymes (e.g., ROS, arginase, NO) that inhibit cytotoxic T cells, dendritic cells, and NK cells [166,167]. MDSCs also promote Treg differentiation and suppress NK cell activity by reducing IL-2 signaling and producing IL-10 [168].

### 4.5 Cancer-associated inflammation

Chronic inflammation supports tumor growth by promoting DNA damage, angiogenesis, and immune suppression. Immune cells like TAMs, neutrophils, and lymphocytes secrete pro-inflammatory cytokines (e.g., IL-6, TNF-α, IL-1β) that activate NF-κB and STAT3 signaling, enhancing tumor proliferation, survival, and metastasis [[169], [170], [171]].

In colorectal cancer, IL-6/STAT3 signaling drives proliferation and immune evasion, while in breast cancer, TNF-α enhances metastasis and cell migration. TAMs, abundant in melanoma and other cancers, secrete MMPs, VEGF, and growth factors that promote tumor survival, invasion, and angiogenesis [172,173]. In lung cancer, TAMs secrete CCL2 to recruit immunosuppressive cells, further promoting tumor progression [171].

Chronic inflammation fosters immunosuppressive TME characteristics, enriching Tregs, MDSCs, and TAMs that suppress cytotoxic T cells and promote immune evasion. This immunosuppressive environment contributes to resistance against immune checkpoint inhibitors, particularly in cancers like pancreatic cancer [172,173].

Chronic inflammation also induces epigenetic changes that impair tumor antigen presentation and CD8^+^ T cell infiltration, as seen in hepatocellular carcinoma [174]. Current strategies aim to selectively target pro-tumor inflammatory pathways while preserving anti-tumor immunity. IL-6 blockade has shown promise in melanoma by disrupting STAT3-mediated inflammation [175]. Additionally, reprogramming M2 TAMs toward an M1-like phenotype may enhance immunotherapy responses, as demonstrated in glioblastoma models [176]. Targeting pro-tumor inflammation while supporting anti-tumor immunity remains a critical therapeutic goal [177].

### 4.6 Pro-tumor intercellular communications

Pro-tumor intercellular communication refers to the exchange of signals between cancer cells and their surrounding environment that facilitates tumor growth and progression [178]. These communications extend beyond simple signaling molecules such as cytokines, growth factors, and exosomes [179]. They play critical roles in helping tumor cells evade immune detection, develop therapy resistance, and sustain growth. Understanding these interactions is essential for uncovering the mechanisms that drive cancer adaptability and heterogeneity [180].

Within the TME, cancer cells establish complex networks with stromal cells, including fibroblasts, immune cells, endothelial cells, and pericytes, often inducing tumor-supportive phenotypes in these non-malignant cells [181,182]. Communication methods include direct cell-cell contact, secretion of signaling molecules (e.g., cytokines, chemokines, and growth factors), release of extracellular vesicles (EVs), and formation of TNTs [183].

CAFs contribute to tumor progression by releasing cytokines such as TGF-β, IL-6, and IL-8, which promote proliferation, migration, immune evasion, and epithelial-mesenchymal transition (EMT). In breast cancer, CAFs drive metastasis and therapy resistance via TGF-β and CXCL12 signaling [184].

Exosomes and microvesicles are key mediators of tumor-stroma communication. These EVs transport oncogenic proteins, RNAs, and DNA fragments to neighboring cells, reprogramming them into tumor-promoting phenotypes [185]. For example, glioblastoma-derived exosomes enriched with EGFRvIII mRNA can activate proliferative signaling in recipient cells. In pancreatic cancer, EVs carrying mutant KRAS DNA reprogram macrophages toward an M2 phenotype, enhancing immune evasion and tumor growth [186]. Additionally, EVs can transfer regulatory RNAs, such as miRNAs and lncRNAs, which modulate gene expression in stromal cells and promote pro-tumorigenic senescence [187]. In prostate cancer, bone stromal cell-derived EVs containing miR-21 and miR-141 influence osteoclast and osteoblast activity, promoting bone metastasis [188].

TNTs provide another direct communication route, enabling the transfer of cytoplasmic content, including proteins, organelles, and mitochondria, between tumor and stromal cells [189]. Under stress, cancer cells can acquire functional mitochondria from stromal cells via TNTs, enhancing oxidative phosphorylation and increasing resistance to oxidative stress and chemotherapy [190]. For instance, in melanoma, CAFs transfer mitochondria to tumor cells, boosting their survival and drug resistance. In ovarian cancer, TNTs facilitate the transfer of functional lysosomes from mesenchymal stem cells to tumor cells, enhancing their metastatic capacity [191].

Within the TME, tumor cells also manipulate immune components to create an immunosuppressive niche. They secrete molecules such as TGF-β, PD-L1, and IL-10 to inhibit T-cell activation and recruit regulatory T cells (Tregs) and myeloid-derived suppressor cells (MDSCs), thus evading immune surveillance [192]. Tumor-associated macrophages (TAMs) further support tumor progression by producing IL-10, VEGF, and matrix metalloproteinases (MMPs), which promote angiogenesis, extracellular matrix remodeling, and invasion. In hepatocellular carcinoma, TAMs release IL-6 and VEGF, stimulating cancer cell proliferation and vascular growth [193].

Emerging evidence also highlights the role of cancer cell–platelet interactions in metastasis. Platelets can shield circulating tumor cells (CTCs) from immune detection and facilitate their adhesion to vascular endothelium during extravasation, promoting metastatic colonization [[189], [190], [191], [192]]. This process involves the binding of platelet selectins to integrins on CTCs. In breast cancer, platelet-coated CTCs exhibit enhanced ability to extravasate and colonize bone tissue [188].

**Fig. 3:**
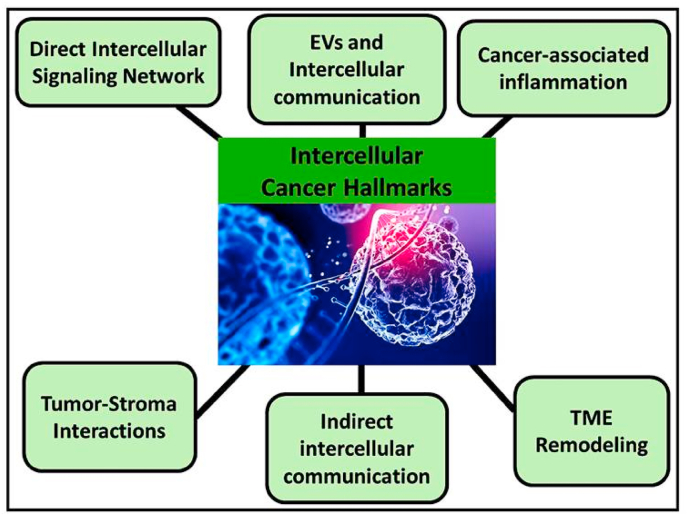
Overview of intercellular tumor hallmarks. Shown are mechanisms of intercellular communication, such as exosome-mediated vesicle exchanges, paracrine signaling, and activation of stromal and immune cells. These processes drive microenvironmental crosstalk and facilitate tumor progression and metastasis.

Targeting intercellular communication in the TME offers promising therapeutic opportunities. Strategies under investigation include inhibiting EV release, blocking TNT formation, and disrupting key cytokine and growth factor signaling pathways [178] Additional approaches aim to repolarize TAMs or prevent CAF activation to convert the TME into an environment that restrains rather than promotes tumor growth [[180], [181], [182], [183], [184], [185],^194^]. Advancing our understanding of these intercellular interactions could lead to more effective therapies that improve cancer outcomes. A summary of tumor-promoting intercellular mechanisms is presented in Fig. 3 and Table 4 illustrating the representative molecular pathways that underpin each hallmark category and proving the dual-rule framework's classification of sites of action and signalling directionality, respectively.

**Table 4:** Summary of intercellular tumor hallmarks. The table highlights intercellular communication pathways that shape tumor progression, including angiogenic signaling, tumor–stroma interactions, pro-inflammatory cytokine signaling, and direct cell–cell adhesion changes. Associated genes and clinical implications demonstrate the relevance of targeting angiogenesis, inflammation, and tumor–stroma crosstalk.

Intercellular Tumor Hallmarks	Mechanism	Key Genes	Preclinical Implications	Clinical Implications	Reference
**Indirect intercellular communication**	VEGF signaling, endothelial cell recruitment	VEGFA, HIF1A, ANGPT1	Enhanced tumor vascularization	Anti-angiogenic therapy (e.g., bevacizumab)	[135,195]
**Cancer-Associated Inflammation**	Pro-inflammatory cytokine production	TNF, IL1B, NFKBIA	Enhanced tumorigenesis	Anti-inflammatory therapy (e.g., NSAIDs)	[106,196]
**Tumor-Stroma Interactions**	CAF-mediated tumor progression	FAP, PDGFRB, TGFBR2	Enhanced tumor growth, invasion	Target for therapy (e.g., FAP inhibitors)	[88,148]
**Direct Intercellular Signaling Network**	Reduced E-cadherin expression	CDH1, CTNNB1, P120	Enhanced tumor progression	Poor prognosis, metastasis	[132,133]
**Tumor Microenvironment Remodeling**	ECM degradation, hypoxia	MMP2, MMP9, HIF1A	Enhanced tumor growth, invasion	Target for therapy (e.g., MMP inhibitors)	[197,198]

## 5 Hallmarks at the extracellular level

### 5.1 Extracellular matrix remodeling

The extracellular matrix (ECM) is a dynamic structural network that plays a crucial role in cancer progression. It contains matrix metalloproteinases (MMPs) that degrade collagen, facilitating tumor invasion, while fibronectin deposition supports the adhesion of circulating tumor cells. ECM remodeling not only promotes metastasis but also regulates the availability of growth factors, such as VEGF, which are essential for tumor proliferation and escape from dormancy [105].

Solid tumors are composed of both cancer cells and the TME, which includes fibroblasts, endothelial cells, immune cells, adipocytes, and the ECM. The ECM provides structural support, regulates tissue homeostasis, and influences cell proliferation [3]. It consists of proteoglycans, glycoproteins, and matricellular proteins, including SPARC, osteopontin (OPN/SPP-1), thrombospondin (THBS/TSP), tenascin, collagen, and laminin [199].

ECM remodeling is characterized by increased collagen deposition and the activation of enzymes such as MMPs, lysyl oxidase (LOX), LOX-like proteins (LOXLs), and WNT1-inducible signaling pathway proteins (WISPs) [166]. The ECM composition varies by tissue type; for example, reticular fibers are common in loose connective tissue, while bone ECM is rich in collagen and minerals [200].

Growth factors, nutrients, and oxygen levels activate signaling pathways such as mTOR through PI3K/AKT. mTORC2 regulates cytoskeletal organization, cell survival, and migration, contributing to tumor invasiveness [201]. Immune cell infiltration into tumors is influenced by ECM remodeling, which affects the recruitment and function of T cells and B cells within the TME [202]. Targeting ECM remodeling enzymes presents potential therapeutic strategies, as these processes are critical for cancer progression and metastasis [197,203]. A comprehensive understanding of ECM composition and structural changes may lead to the identification of novel diagnostic markers and therapeutic targets [198,204].

### 5.2 Extracellular enzymes mediated alterations

Extracellular enzymes secreted by tumor and stromal cells play key roles in modifying the ECM, shaping immune responses, and supporting cancer cell survival and metastasis (3, 91). These enzymes mediate direct interactions between cancer cells and other TME components, including immune cells, fibroblasts, adipocytes, and vascular cells, facilitating tumor progression through protein-protein interactions and secreted signaling molecules [205].

Matrix Metalloproteinases (MMPs): Members of the M10 protease family, including 17 secreted and 6 membrane-associated types. MMPs degrade ECM components and release pro-angiogenic factors, supporting invasion and metastasis [206]. Serine Proteases: Proteins such as trypsin and chymotrypsin that degrade ECM and activate MMPs, facilitating tumor cell invasion [207]. Cysteine Proteases are ndopeptidases that contribute to ECM degradation and promote angiogenesis, metastasis, and tumor invasion [195]. Aspartic Proteases are involved in ECM breakdown, angiogenesis, metastasis, and resistance to apoptosis [208].

These enzymes also release ECM-sequestered growth factors, such as FGF2, amplifying tumor-promoting signals. Given their roles in cancer progression, extracellular enzymes are potential biomarkers and therapeutic targets [205].

### 5.3 Extracellular vesicle (EV) mediated changes

EVs are nano-sized membrane-bound particles secreted by both normal and cancer cells. EVs facilitate intercellular communication by transporting bioactive molecules, including proteins, RNAs, and lipids, which influence tumor growth, metastasis, and resistance to therapy [87].

EVs are heterogeneous and include subtypes such as exosomes, large oncosomes, migrasomes, ectosomes, exomeres, supermeres, apoptotic blebs, and membrane particles [185]. They are present in various biological fluids, including blood, urine, cerebrospinal fluid, and saliva, under both healthy and diseased conditions [209]. EVs contribute to pre-metastatic niche formation, immune evasion, and therapy resistanceby facilitating drug resistance and promoting tumor heterogeneity [210].

Exosomes originate from multivesicular bodies, while microvesicles bud directly from the plasma membrane [211]. EVs are taken up by recipient cells through mechanisms such as endocytosis, membrane fusion, or receptor-mediated signaling, altering cellular phenotypes. Due to their stability and disease-specific molecular contents, EVs are being investigated as diagnostic and prognostic biomarkers, as well as potential drug delivery vehicles [199,212].

### 5.4 Extracellular small DNAs and RNAs

Extracellular small DNAs (exDNA) and RNAs (exRNA) are nucleic acid fragments present in the extracellular space, playing significant roles in intercellular communication and cancer progression [87]. These molecules contribute to tumor growth, metastasis, immune evasion, and therapy resistance, making them valuable as potential biomarkers and therapeutic targets [3]. ExDNA and exRNA are found in body fluids such as serum, urine, cerebrospinal fluid, lymph, bile, and breast milk, and are detectable through liquid biopsy techniques, offering minimally invasive cancer diagnostics and monitoring [213,214]. Generation of exDNA and exRNA is contributed due to active release by neutrophil extracellular traps (NETs) and passive release due to cell death processes such as apoptosis, necrosis, and lysis of cancer cells (9, 63). ExRNAs regulate gene expression in cancer, influencing cell proliferation and survival. For instance, exosomal miRNAs such as miR-21 and miR-155, and lncRNAs, are often elevated in cancer patients and are stable in biological fluids, making them suitable biomarkers [215]. Further research into extracellular nucleic acids may provide new avenues for early diagnosis, treatment monitoring, and targeted therapy in cancer.

### 5.5 Extracellular metabolites of local niches of tumor

The TME is rich in extracellular metabolites that play critical roles in adipogenesis, cancer progression, and cellular behavior. These metabolites, secreted by tumor and stromal cells, contribute to metabolic adaptability, immune evasion, and tumor heterogeneity [9]. Understanding their dynamics offers insight into the metabolic interplay that supports malignancy [3].

Key extracellular metabolites involved in cancer progression include amino acids, amines, lipids, carbohydrates, and adenosine [216]. For example, glutamine at elevated concentrations supports anabolic processes such as nucleotide and protein synthesis, while accumulated free fatty acids (FFAs) enhance β-oxidation, providing energy under metabolic stress [83,217]. Oncometabolites, such as 2-hydroxyglutarate (2-HG) produced by IDH1/2 mutations, disrupt epigenetic regulation by inhibiting α-ketoglutarate-dependent dioxygenases, leading to abnormal gene expression that promotes tumorigenesis [218]. Additionally, increased adenosine levels in hypoxic TMEs suppress anti-tumor immunity by inhibiting T-cell activation and promoting regulatory T-cell (Treg) activity [219].

Polyamines, including spermidine and putrescine, further support tumor growth, and immune suppression by impairing T-cell function [220]. Although these metabolites strongly drive tumorigenesis, they often reflect the influence of the TME rather than intrinsic cancer metabolism [88]. Therefore, targeting these key metabolites and their regulatory pathways presents a promising therapeutic strategy [221].

### 5.6 Exogenous cytokines and hormones

Exogenous cytokines and hormones significantly influence tumor growth, immune modulation, and metastasis. Their signaling can either promote or suppress tumor progression, depending on the context, adding complexity to tumor heterogeneity [3]. Both cytokines and hormones are often overproduced or dysregulated in the TME, contributing to tumorigenesis.

Key pro-tumorigenic cytokines include IL-6, TNF-α, TGF-β, chemokines (CKs), and interferons (IFNs). IL-6 promotes tumorigenesis via the STAT pathway in cancers such as breast, lung, and colorectal cancer [196]. TNF-α facilitates epithelial-mesenchymal transition (EMT), while TGF-β supports immune evasion, angiogenesis, and resistance to apoptosis. Chemokines and IFNs also contribute to cancer progression and angiogenesis [222,223].

Hormones such as estrogen, progesterone, androgens, and insulin further promote tumor growth. Estrogen influences breast, ovarian, and uterine cancers by activating MAPK and PI3K/AKT pathways via estrogen receptors (ERs) [223]. Progesterone activates cyclin D1 pathways through progesterone receptors (PRs), and androgens act through androgen receptors (ARs) to stimulate growth-promoting cascades like the PI3K/AKT and MAPK pathways [103]. Insulin and insulin-like growth factor (IGF) activate PI3K/AKT/mTOR and Ras/MAPK pathways via insulin receptors (IRs), supporting carcinogenesis and angiogenesis, particularly in hyperinsulinemic states [224]. Targeting these cytokines and hormones may offer effective therapeutic options to disrupt tumor growth, metastasis, and immune evasion [173].

### 5.7 Inducing angiogenesis

Tumor angiogenesis, primarily driven by vascular endothelial growth factor (VEGF) and hypoxia, ensures nutrient supply and supports metastasis. The “angiogenic switch” marks the transition to a pro-angiogenic state that fuels tumor progression [225]. Understanding its contribution to tumor heterogeneity is essential for developing targeted therapies [3,222].

Angiogenesis in tumors involves sprouting, glomeruloid, and intussusceptive microvascular growth, regulated by a balance of pro-angiogenic and anti-angiogenic factors. Hypoxia stabilizes hypoxia-inducible factor 1 (HIF-1), which induces angiogenesis-related genes such as VEGF, bFGF, and PDGF [226]. VEGF isoforms, including VEGF-A, VEGF-B, VEGF-C, and VEGF-D, have distinct roles. For example, VEGF-A promotes the formation of tip cells necessary for sprouting angiogenesis [227].

Anti-angiogenic therapies, such as VEGF inhibitors, face limitations due to compensatory pathways. Combining these therapies with adjuvants may improve efficacy and prevent resistance and recurrence [228]. Biomarkers like angiopoietins, VEGF-A, EGFL7, and PECAM-1/CD31 are valuable for monitoring therapeutic response [229].

Ultimately, the vascular remodeling that supports angiogenesis paves the way for cellular dissemination, culminating in invasion and metastasis.

### 5.8 Activating invasion and metastasis

Invasion and metastasis are critical hallmarks of cancer, enabling tumor cells to spread from primary sites to distant organs, often leading to treatment failure and poor prognosis [204]. This process involves genetic and epigenetic changes, extracellular matrix (ECM) remodeling, and dynamic interactions with the TME (3).

Cancer cells invade surrounding tissues and metastasize via a multistep cascade. EMT is central to this process, during which epithelial cells lose adhesion and acquire mesenchymal traits, enhancing their migratory capabilities [85]. EMT is associated with the downregulation of adhesion molecules like E-cadherin and claudins and the upregulation of mesenchymal markers like fibronectin and MMP-9 [[230], [231], [232]]. ECM degradation further facilitates cancer cell migration.

During intravasation, cancer cells enter the bloodstream by traversing endothelial barriers. They subsequently adhere to distant endothelial cells and extravasate to colonize secondary sites [233]. Non-cancerous cells within the TME contribute to invasion and metastasis through paracrine signaling and immune modulation [87].

**Fig. 4:**
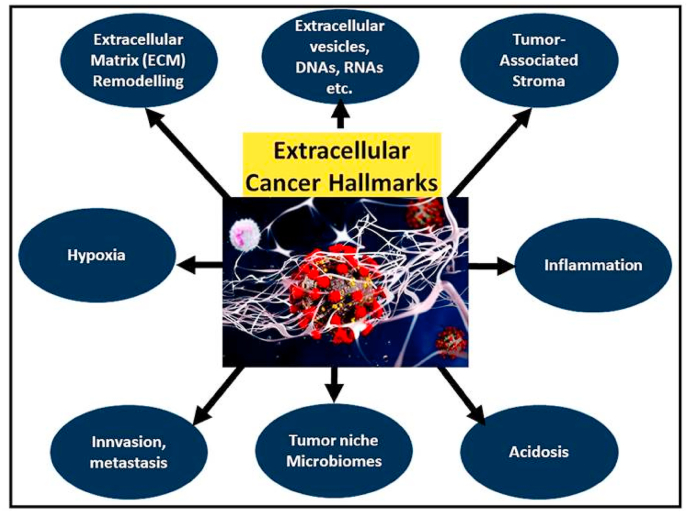
Representation of extracellular tumor hallmarks. The figure depicts the roles of extrachromosomal circular DNA (eccDNA), matrix metalloproteinases (MMPs), and extracellular matrix remodeling in sustaining tumor invasion, angiogenesis, and therapy resistance.

Therapies targeting EMT, ECM degradation, and cell-cell communication are potential strategies to prevent metastasis and improve outcomes [234]. A list of hallmark definitions and related molecular determinants is provided in Table 5, within the dual-rule framework, Fig. 4 illustrates site-of-action classification and signaling directionality.

**Table 5:** Summary of extracellular tumor hallmarks. Key extracellular-level processes are outlined, including ECM remodeling, stromal activation, inflammation, acidosis, hypoxia, invasion, and angiogenesis. Relevant genes and proteins are listed alongside their implications in tumor progression, with therapeutic opportunities such as MMP, VEGF, HIF1A, and LDHA inhibitors.

Extracellular Tumor Hallmarks	Mechanism	Key Genes	Preclinical Implications	Clinical Implications	Reference
**Extracellular Matrix (ECM) Remodeling**	ECM degradation, stiffness increase	MMP2, MMP9, COL1A1	Enhanced tumor growth, invasion	Poor prognosis, metastasis	[193,201]
**Tumor-Associated Stroma**	CAF-mediated tumor progression	FAP, PDGFRB, TGFBR2	Enhanced tumor growth, invasion	Target for therapy (e.g., FAP inhibitors)	[147,191]
**Inflammation**	Pro-inflammatory cytokine production	TNF, IL1B, NFKBIA	Enhanced tumorigenesis	Anti-inflammatory therapy (e.g., NSAIDs)	[163,168,192,200]
**Acidosis**	Increased lactate production, pH reduction	LDHA, MCT1, CA9	Enhanced tumor growth, invasion	Target for therapy (e.g., LDHA inhibitors)	[3,11]
**Activating invasion and metastasis**	Overexpression of MMPs, VEGFA EPCAM	MMPs, VEGFA EPCAM	Enhanced invasion and spread of tumor	Target for therapy (e.g. MMP and VEGF inhibitors	[205,206]
**Hypoxia**	HIF1A-mediated transcriptional regulation	HIF1A, VEGFA, GLUT1	Enhanced tumor growth, invasion	Target for therapy (e.g., HIF1A inhibitors)	[103,104]
**Angiogenesis**	VEGF signaling, endothelial cell recruitment	VEGFA, HIF1A, ANGPT1	Enhanced tumor vascularization	Anti-angiogenic therapy (e.g., bevacizumab)	[103,222,225]

## 6 Technological advances: Tools shaping the expanding hallmarks

Single-cell omics, spatial transcriptomics, and AI-driven modeling now resolve tumor heterogeneity at unprecedented resolution. These tools identify actionable targets (e.g., metabolic vulnerabilities) and optimize combination therapies [[233], [234], [235], [236]]. Important technologies, which are illustrated below, have advanced the understanding of the cellular diversity of the TME and thereby expanded the hallmarks framework. Multiplexed Imaging Techniques: Advances in imaging technology, such as multiplexed immunofluorescence and spatial transcriptomics, have now allowed the visualization and quantification of the spatial arrangement of distinct cell types present within the TME, providing valuable insights into the spatial distribution of cancer stem cells, immune cells, and stromal cells [[237], [238], [239], [240], [241]]. The spatial distribution of signaling molecules involved in interactions within the TME can be simulated by mathematical modeling frameworks, thereby giving information on gradients formed across the TME and providing insight into the type of cellular diversity it harbors. This further aids in predicting the behavior of cell populations, such as cancer stem cells (CSCs) with varying EMT phenotypes [235].

The use of high-throughput approaches in proteomics and genomics has made it easier to identify important signaling pathways and molecular mechanisms responsible for cellular heterogeneity in the TME. The molecular basis of cellular heterogeneity and how it affects tumor characteristics can be unraveled by examining gene expression and protein levels in unique cell populations. Therefore, advances like exome, transcriptome, DNA methylation, and proteome profiling in cancer research are critical to understanding the concept of tumor hallmarks [236]. Data integration in spatial transcriptomics reveals that the knowledge of complex phenotypic properties of cancer cells alongside their microenvironment contributes to the spatial contribution of all tumor compartments to their hallmarks in cancer. The technology involved in such an approach, especially proteogenomics and network-based data analysis, inherently offers a holistic view of tumor hallmarks by considering cancer as a disease of the cellular system rather than the genome [237].

Multi-omics techniques include the integration of technologies such as genomics, transcriptomics, proteomics, metabolomics, and epigenomics. Through these approaches, complex biological systems like cancer can be interpreted to gain a clear perspective of their molecular information content [238]. Simultaneous examination of different layers of molecular data identifies significant molecular markers associated with the onset of cancer and elucidates the challenging relationships of various biological components involved [239]. It further enables a more robust characterization of the molecular landscape of tumors, cancer subtypes, disease progression, and treatment response, which has been used to identify new biomarkers, therapeutic targets, and personalized treatment approaches through a holistic approach. A summary of potential biomarkers and their involved molecular targets is presented in Table 6, which draws attention to the therapeutic targets and translational implications associated with each hallmark level.

**Table 6:** Emerging tumor biomarkers with key molecular pathways and potential clinical applications based on the cellular, intracellular, intercellular, and extracellular hallmark.

Emerging Marker/Mechanism	Framework Level	Key Molecule/Pathway	Evidence Strength	Drugability/Investigational Application	Representative References
Extrachromosomal circular DNA (eccDNA)	Intracellular	c-Myc amplification, chromothripsis	In vitro, patient-derived tumor sequencing	Potential biomarker for genomic instability and therapy resistance	(50–54, 160)
Mitochondria–ER cross-talk	Intracellular → Cellular	Ca^2+^ flux, unfolded protein response (UPR)	In vitro, in vivo models	Targeting Ca^2+^ transporters, ER stress modulators under investigation	(120–122)
Immunometabolic regulation	Cellular	PD-1/PD-L1, glycolysis–OXPHOS switch	Clinical trial correlates, in vivo models	Immune checkpoint inhibitors; metabolic modulators (e.g., IDO inhibitors)	(130–132)
Extracellular vesicles (EVs)	Intercellular/Extracellular	Exosomal miRNA-21, PD-L1 cargo	In vitro, in vivo, clinical biomarker studies	Diagnostic assays in liquid biopsy; EV-targeting strategies in trials	(185–187)
Cytokine-mediated immune suppression	Intercellular	IL-6/JAK/STAT3, TGF-β/SMAD	In vivo, clinical association studies	Anti-IL-6R (tocilizumab), TGF-β inhibitors (investigational)	(87–88)

Artificial intelligence platforms are increasingly applied to integrate complex multi-omics datasets and reveal clinically actionable insights. Tools such as MOFA + (Multi-Omics Factor Analysis) and DeepMOA enable latent factor discovery across transcriptomic, epigenomic, and proteomic layers [240]. AI-driven proteogenomic networks, exemplified by DeepCC, AutoOmics, and Cell2location stratify patients based on molecular signatures and predict therapy resistance patterns [241]. Such pipelines demonstrate how artificial intelligence can integrate diverse multi-omics datasets to provide a high-resolution view of tumor biology. scMultiome simultaneously captures single-cell chromatin accessibility and gene expression, enabling identification of regulatory programs driving tumor heterogeneity. DeepMOFA applies factor analysis to combine transcriptomic, epigenomic, and proteomic layers, uncovering latent structures that define tumor subpopulations and their functional states [240]. Cell2location maps cell types within spatial tissue contexts, revealing interactions between immune and tumor cells that guide immune evasion and therapeutic response [241]. Together, these tools allow precise delineation of tumor sub-niches, cellular heterogeneity, and patient-specific vulnerabilities, supporting predictive modeling for targeted and immunotherapies. These approaches are now being piloted to optimize biomarker panels for liquid biopsy platforms, inform patient stratification for immunotherapy, and guide adaptive trial designs in precision oncology [242].

Data integration is critical in comprehending complex networks and interactions that control biological systems in systems biology. All-inclusive models that capture the complexity of cancer biology can be developed by integrating diverse omics data with clinical and histopathological information [243]. This integrated data will enable the identification of molecular networks, signaling pathways, and regulatory mechanisms that enhance carcinogenesis and alter therapy outcomes. Technical advances in recent years have made it possible to study tumors at a level of detail previously unimaginable, particularly in spatial transcriptomics [244]. This enables researchers to map at single-cell resolution and represent the geographic distribution of numerous cell types within tumors. These advanced technologies allow us to gain insights into the spatial Niche-specific adaptation of cancer cells, reveal complex cellular interactions within the TME, and identify regionally varying activities of cancer hallmarks [245]. This extensive approach to tumor hallmarks offers improved comprehension of the spatial nature of tumors, providing deep knowledge for the development of medicines specifically targeted to the characteristics of particular tumors and for individualized treatment plans [246].

## 7 Future perspectives: fighting the complexity of expanding hallmarks of cancer

The hallmarks of cancer continue to evolve with discoveries that extend beyond uncontrolled proliferation and apoptosis evasion, now encompassing immune modulation, inter-organelle communication, metabolic adaptation, and neuronal mimicry [3]. Emerging strategies like synthetic lethality—where tumors rely on specific gene interactions, offer promising avenues for precise, low-toxicity treatments [247,248]. Identification of synthetic lethal gene pairs, such as those linked to BRCA1/2 mutations, has facilitated the development of personalized therapies based on genetic vulnerabilities [249]. Understanding and targeting these emerging mechanisms will be essential for comprehensive and durable cancer treatment [84].

### 7.1 Emerging approaches in targeting TME dynamics

Therapeutic strategies increasingly focus on modifying the TME, particularly by targeting tumor-associated macrophages (TAMs) and myeloid-derived suppressor cells (MDSCs), which facilitate immune evasion and tumor progression [168]. Current approaches aim to reprogram TAMs toward anti-tumor phenotypes and inhibit MDSC expansion to enhance immunotherapy [179]. Nanomedicine-based delivery of immunomodulatory agents to TAMs has shown promise in preclinical models, particularly using lipid-based nanoparticles carrying Toll-like receptor agonists [250,251].

Additionally, EVs are now recognized as key mediators of cell-cell communication in the TME, contributing to tumor growth and drug resistance [182]. Therapeutically targeting EVs or their cargo offers a novel approach to disrupt tumor-supportive networks [199,252].

### 7.2 Immunometabolism and precision oncology: novel therapeutic strategies

#### 7.2.1 Immunometabolism and cancer treatment

Immunometabolism, which examines metabolic interactions between cancer and immune cells, represents a significant frontier in cancer therapy. Cancer cells often manipulate metabolic pathways to suppress immune responses. For example, inhibition of indoleamine 2,3-dioxygenase (IDO)—an enzyme involved in tryptophan metabolism—can counteract immunosuppression and enhance anti-tumor immunity. Early-phase clinical trials combining IDO inhibitors with immune checkpoint blockade have shown encouraging results [253,254]. Targeting tumor metabolism, such as lactate production via lactate dehydrogenase A (LDHA) inhibition, is another promising strategy to alleviate immunosuppressive TME conditions [255,256]. These approaches are advancing the field of precision oncology by exploiting cancer-specific metabolic dependencies.

Tumor cell and infiltrating immune cell metabolic rewiring creates an immunosuppressive TME. Glycolytic, oxidative phosphorylation, and amino acid metabolism changes suppress anti-tumor immunity and are responsible for resistance to checkpoint inhibitors [257]. Metabolic markers like LDHA, IDO, and arginase can be used as clinical biomarkers for immune fitness, and metabolic imaging tools like FDG-PET can provide surrogate markers for treatment monitoring [258]. Combination of metabolic and immune biomarkers also promises potential for companion diagnostics directing immunotherapy stratification [259].

### 7.3 Contribution of artificial intelligence to hallmarks of cancer

The integration of artificial intelligence (AI) and machine learning is transforming cancer detection, classification, and treatment personalization. AI-driven analyses of multi-omics datasets are uncovering hallmark-specific biomarkers and refining therapeutic target selection. Deep learning algorithms predict patient-specific treatment responses and potential resistance mechanisms, supporting individualized oncology strategies [260,261]. AI is also optimizing clinical trial designs by identifying probable responders to hallmark-targeted therapies [129]. In imaging, AI enhances the detection of hallmark features, such as metabolic alterations and immune evasion, using non-invasive techniques [108].

### 7.4 Targeting inter-organellar communications

Cancer cells exploit inter-organelle communication pathways to maintain survival under stress. For example, the endoplasmic reticulum (ER)-mitochondria axis, mediated by MAMs, regulates calcium signaling, lipid metabolism, and reactive oxygen species (ROS) balance, supporting tumor cell viability [130]. Disruption of these interactions can induce apoptosis, as shown in glioblastoma models [262]. Lysosomes also play a crucial role in metabolic adaptation through autophagy, and inhibitors of lysosomal function are under investigation for cancers with high autophagic activity, such as pancreatic cancer [263]. Future studies must address the compensatory mechanisms within these communication networks to design effective therapeutic strategies [3].

The future of cancer therapy lies in addressing the growing complexity of tumor hallmarks. Strategies that target the TME, immunometabolism, inter-organelle communication, and leverage AI for precision medicine are shaping next-generation cancer treatments [129,130,[261], [262], [263], [264], [265], [266]]. These approaches promise more personalized, durable, and effective therapies by directly confronting the adaptability and heterogeneity of tumors.

Future translational oncology directions can be summarized into three testable roadmaps. TME remodeling aims to overcome resistance to immunotherapy by interfering with stromal and immune interaction [267]; spatial transcriptomics, multiplex IHC, and co-culture models can measure cytokine signatures like TGF-β, IL-6, and CXCL12, where basket trials are testing FAP inhibitors or TGF-β blockers in combination with checkpoint inhibitors [268] and companion diagnostics from stromal markers. Second, immunometabolism and synthetic vulnerabilities touch on how metabolic rewiring drives resistance; single-cell metabolomics and CRISPR-based synthetic lethality screens can determine dependencies that include LDHA, IDO, arginase, and glutamine transporters, with IDO or glutamine inhibitors and adaptive trials combined with immunotherapies and stratified by metabolic signatures. Third, AI-driven multi-omics integration strives to create predictive biomarkers and optimize patient stratification [269]; deep learning on spatial transcriptomics, proteomics, imaging, and ctDNA can provide composite signatures like eccDNA amplifications (EGFR, MYC) [270], immune-stromal interaction scores, and EV cargo (miRNA-21, PD-L1) [271], with umbrella trials evaluating AI-driven biomarker allocation and dynamic algorithm recalibration. Together, these roadmaps turn conceptual progress into practice, bridging research questions with methods, indicators, and trial designs.

### 7.5 Translational challenges and clinical implementation

The main challenge is validation of suggested biomarkers in large, multicentre, prospective cohorts that are representative of the diversity of the population. Harmonized sampling either from tissue, blood, or EVs, must be achieved for reproducibility. Standardized pre-analytical procedures must be implemented to avoid batch effects and technical variability. Without such attention to rigor, numerous biomarker signatures do not advance beyond initial stages of discovery.

Complex diagnostics, especially those that are multi-analyte or AI-supported, must contend with strict regulatory environments [267]. Model governance needs to be transparent to a greater extent, from versioning the algorithms to checking for drift and making sure of explainability [267]. Clinical performance metrics need to go beyond the AUROC values to draw calibration curves, decision-curve analyses, and real-world net benefit evaluations [272]. All these demands, though time-consuming, are necessary for patient safety and trust in new technology.

State-of-the-art methods like spatial transcriptomics, single-cell multi-omics, and high-throughput proteomics have revolutionized cancer biology, but are still expensive and technically challenging [273]. Their applications are now restricted to high-end research institutions. Wider dissemination will need technological miniaturization, affordability, and incorporation into standard pathology pipelines. Without this, access inequities between high-resource and low-resource environments could be exacerbated [273].

Today's clinical practice is too often based on single biomarkers, but the complexity of cancer requires composite signatures combining tumor-intrinsic attributes (genomic alterations, epigenomic changes, metabolic rewiring) with tumor-extrinsic influences (TME composition, cytokine milieu, EV cargo) [[274], [275], [276]]. Multi-dimensional models of this sort will succeed clinically only if complemented by predefined cut-offs, therapy-selection algorithms, and prospective validation studies [[277], [278], [279], [280], [281], [282]].

Implementation science. Just as significant is the application of findings to standard oncology practice [[279], [280], [281], [282]]. Biomarker-guided tests need to be integrated into clinical practices with consideration of turnaround times, reimbursement models, and cost-benefit analysis [[278], [279], [280]]. Educational programs need to train clinicians and pathologists to interpret multi-omics results and AI-derived risk scores [[281], [282], [283]]. Implementation science provides the assurance that these advances are translated from academic discourse to actionable agents that benefit patients [[279], [280], [281], [282]].

Translating the evolving framework of tumor hallmarks into clinical practice invites both futuristic opportunities and realistic challenges. On the futuristic side, advances such as spatial transcriptomics, single-cell multi-omics, and AI-driven analytics promise to decode tumor heterogeneity with unprecedented resolution, enabling the development of truly personalized therapeutic strategies [16,[240], [241], [242]]. Novel concepts, including synthetic lethality, extracellular vesicle–mediated signaling, and inter-organelle crosstalk, provide actionable entry points for therapeutic innovation [127,138,199], while liquid biopsy platforms analyzing eccDNA or exRNA offer minimally invasive avenues for continuous monitoring of clonal evolution and therapy response [59,[213], [214], [215]]. These approaches have the potential to fundamentally reshape diagnostics, prognostics, and treatment paradigms in oncology.

Yet, several realistic challenges constrain translation. The intrinsic heterogeneity of tumors across patients, tumor sites, and temporal stages complicates biomarker validation and restricts the universal applicability of therapeutic targets [[42], [43], [44], [45],^145^]. Integrating multi-omics datasets into clinically actionable insights requires standardization, reproducibility, and large-scale longitudinal validation, all of which remain resource and time-intensive [239,243]. Additionally, adaptive resistance mechanisms, often mediated by compensatory pathways, undermine the long-term efficacy of targeted interventions [[23], [24], [25], [26], [27],^75^]. Ethical, regulatory, and infrastructural barriers particularly surrounding AI-guided decision-making and high-cost precision platforms further complicate implementation, while disparities in healthcare access raise concerns about global equity [242,246]. Addressing these translational challenges will require integrative validation pipelines, cross-disciplinary collaborations, and a balance between technological innovation and accessibility.

**Fig. 5:**
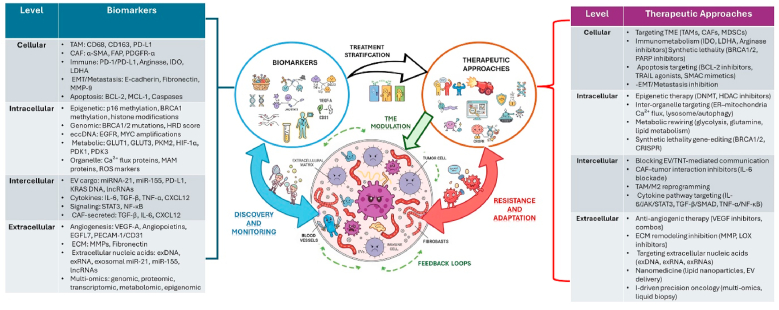
A summary on Integration of tumor hallmarks with clinical prospects. This schematic links fundamental cancer hallmarks with clinical applications, highlighting implications for prognosis, biomarker development, therapeutic targeting, and personalized oncology.

Collectively, these translational problems underscore the disconnect between conceptual paradigms and bedside implementation. Solving them in an orderly fashion will dictate whether the growing universe of tumor hallmarks can be converted into functional diagnostic, prognostic, and therapeutic applications. A summarized model on the rationale of cellular, intracellular, intercellular and extracellular hallmarks in the contexts of TME, diagnostic and therapeutic avenues is illustrated that summarizes key technologies, biomarkers, and applications for studying tumor heterogeneity, TME dynamics, and therapy response, highlighting their translational relevance in precision oncology (Fig. 5 and Table 7).

**Table 7:** Summarizes key technologies, biomarkers, and applications for studying tumor heterogeneity, TME dynamics, and therapy response, highlighting their translational relevance in precision oncology.

Aim	Method	Biomarker	Application	References
Map spatial heterogeneity of tumor/TME	Multiplexed Immunofluorescence, Spatial Transcriptomics	CSCs (CD44, ALDH1), immune markers (CD8, FOXP3), niche-specific genes	Visualize tumor sub-niches, EMT states, immune-stromal interactions	[[235], [236], [237], [238], [239], [240], [241],^244^,^245^]
Resolve regulatory programs in single cells	scMultiome, DeepMOFA	Chromatin accessibility, RNA expression, latent omics factors	Identify tumor subpopulations & functional states	[240]
Map cell types in tissue context	Cell2location	Immune-tumor spatial interactions	Predict immune evasion & therapy response	[241]
Predict therapy response & resistance	AI-driven Proteogenomics (DeepCC, AutoOmics)	Molecular signatures	Patient stratification, optimize treatment	[241,242]
Track tumor evolution non-invasively	Liquid Biopsy (ctDNA, eccDNA, exRNA)	EGFR, MYC, PD-L1, miRNA-21	Monitor clonal evolution, guide adaptive therapy	[59,[213], [214], [215],^270^,^271^]
Target immunometabolism & TME	Metabolic biomarkers, Nanomedicine, EV targeting	LDHA, IDO, arginase, TAMs, MDSC, EV cargo	Companion diagnostics, TME reprogramming, immunotherapy	[182,199,[250], [251], [252], [253], [254], [255], [256], [257], [258], [259]]

Ultimately, this framework redefines the frontiers of precision oncology, seamlessly integrating multi-omics, spatial, and single-cell technologies to translate intricate tumor heterogeneity into actionable, patient-specific strategies. By connecting cellular, molecular, and microenvironmental hallmarks with AI-driven analytics and predictive modeling, it establishes an unparalleled roadmap for cross-omics integration, guiding the discovery of novel biomarkers, therapeutic targets, and adaptive treatment designs. This approach not only empowers clinicians to tailor interventions with unprecedented precision but also propels the field toward a future where the complexity of cancer is not just understood, but strategically exploited to achieve truly personalized, next-generation cancer care.

## 8 Conclusion

The expanding hallmarks of cancer underscore the disease's adaptability, driven by genetic, metabolic, and microenvironmental factors across intracellular, cellular, intercellular, and extracellular levels. Targeting these interconnected traits using OMICS-based technologies, immune-metabolic strategies, synthetic lethality, and AI-guided precision medicine is central to developing next-generation therapies. A comprehensive understanding of tumor characteristics-such as metabolic deregulation, immune evasion, and intercellular communication, across these biological layers may help address the complexity of cancer.

Computational modeling can further aid in predicting tumor evolution and therapy responses by simulating dynamics at multiple biological scales. This integrated approach could inform personalized treatment strategies and help answer key questions, such as the role of mitochondrial transfer via TNTs in metastasis, or the design of clinical trials that simultaneously target metabolic, immune, and epigenetic pathways.

A refined framework for understanding tumor hallmarks may also enhance the precision of diagnostic marker identification, particularly when such markers are enriched in specific tumor compartments. AI can play a critical role in analyzing the dynamics of biomolecules, including DNA, proteins, microRNAs, circular RNAs, extrachromosomal DNA, and exosomal components, across intracellular, intercellular, and extracellular spaces, supporting more accurate and individualized cancer management.

While core hallmarks such as apoptosis resistance, sustained angiogenesis, and uncontrolled proliferation remain central to cancer biology, emerging hallmarks, including immunometabolic modulation, inter-organelle communication, metabolic reprogramming, and TME crosstalk, highlight cancer as a highly dynamic, adaptable, and evolving disease. These layers of complexity demand multi-dimensional therapeutic strategies.

The increasing focus on intercellular signaling, metabolic shifts, and TME interactions is driving the development of more targeted and adaptable cancer treatments. AI is expected to further revolutionize oncology by improving biomarker discovery, optimizing treatment selection, enabling early detection, and predicting therapeutic outcomes with high precision. Additionally, gene editing tools like CRISPR, synthetic lethality-based drug targets, nanomedicine, and immune-modulating therapies can be specifically designed to target tumor hallmarks at distinct biological levels whether intracellular, cellular, intercellular, or extracellular.

This review comes back to the canons of cancer in the context of new evidence bringing to the fore an increase of complexities over traditional models. We advance a hierarchical view, cutting across intracellular, cellular, intercellular, and extracellular levels, which incorporates understudied dimensions including eccDNA, cross-organelle communication, immunometabolic regulation, and extracellular vesicle signaling. Through spatial and single-cell multi-omics and AI-assisted integration, this integration transcends descriptive overviews to produce testable hypotheses, clinical markers, and translational blueprints. The goal is to lead researchers from mechanistic understanding towards actionable strategies in precision oncology.

## Declaration of generative AI and AI-assisted technologies in the writing process

During the preparation of this work the author(s) used [Generative AI Chatbots] to [improve the language]. After using this tool/service, the author(s) reviewed and edited the content as needed and take(s) full responsibility for the content of the publication.

## CRediT authorship contribution statement

**Hasmiq L. Arora:** Conceptualization, Data curation, Writing – original draft. **Gopinath Sekar:** Conceptualization, Data curation, Formal analysis, Writing – original draft. **Anushka Phadnis:** Data curation, Formal analysis, Writing – original draft. **Anjali Bahot:** Formal analysis, Writing – original draft. **Dhanashree Bomle:** Conceptualization, Data curation, Writing – original draft. **Vaidehi Patel:** Conceptualization, Writing – original draft. **Jayanta K. Pal:** Writing – original draft, Writing – review & editing. **Sachin C. Sarode:** Conceptualization, Writing – original draft, Writing – review & editing. **Nilesh Kumar Sharma:** Conceptualization, Data curation, Funding acquisition, Investigation, Methodology, Writing – original draft, Writing – review & editing.

## Declaration of competing interest

The authors declare that they have no known competing financial interests or personal relationships that could have appeared to influence the work reported in this paper.

## Data Availability

No data was used for the research described in the article.
